# Epitopes for Multivalent Vaccines Against *Listeria, Mycobacterium* and *Streptococcus* spp: A Novel Role for Glyceraldehyde-3-Phosphate Dehydrogenase

**DOI:** 10.3389/fcimb.2020.573348

**Published:** 2020-10-28

**Authors:** Carmen Alvarez-Dominguez, David Salcines-Cuevas, Héctor Teran-Navarro, Ricardo Calderon-Gonzalez, Raquel Tobes, Isabel Garcia, Santiago Grijalvo, Alberto Paradela, Asunción Seoane, Felix J. Sangari, Manuel Fresno, Jorge Calvo-Montes, I. Concepción Pérez Del Molino Bernal, Sonsoles Yañez-Diaz

**Affiliations:** ^1^ Grupo de Oncología y Nanovacunas, Instituto de Investigación Marqués de Valdecilla, Santander, Spain; ^2^ Facultad de Educación, Universidad Internacional de La Rioja, Logroño, Spain; ^3^ Independent Researcher, Granada, Spain; ^4^ Biomedical Research Networking Center in Bioengineering, Biomaterials and Nanomedicine (CIBER-BBN), Barcelona, Spain; ^5^ CIC biomaGUNE, Basque Research and Technology Alliance (BRTA), Donostia-San Sebastian, Spain; ^6^ Department of Surfactants and Nanobiotechnology, Institute for Advanced Chemistry of Catalonia (IQAC-CSIC), Barcelona, Spain; ^7^ Centro Nacional de Biotecnología, CSIC, Madrid, Spain; ^8^ Department of Molecular Biology, Instituto de Biomedicina y Biotecnologia de Cantabria (IBBTEC, CSIC-Universidad de Cantabria-SODERCAN), Santander, Spain; ^9^ Facultad de Medicina, Universidad de Cantabria, Santander, Spain; ^10^ Department of Molecular Biology, DIOMUNE S.L., Madrid, Spain; ^11^ Centro de Biología Molecular Severo Ochoa, Universidad Autónoma de Madrid, Madrid, Spain; ^12^ Servicio de Microbiología, Hospital Universitario Marqués de Valdecilla, Santander, Spain; ^13^ Servicio de Dermatología, Hospital Universitario Marqués de Valdecilla, Santander, Spain; ^14^ Departamento de Medicina y Cirugia, Facultad de Medicina, Universidad de Cantabria, Santander, Spain

**Keywords:** adjuvants, glyceraldehyde-3-phosphate-dehydrogenase, listeriosis, pneumonia, tuberculosis, vaccines

## Abstract

The glycolytic enzyme and bacterial virulence factor of *Listeria monocytogenes*, the glyceraldehyde-3-phosphate dehydrogenase (GAPDH, Lmo2459), ADP-ribosylated the small GTPase, Rab5a, and blocked phagosome maturation. This inhibitory activity localized within the NAD binding domain of GAPDH at the N-terminal 1–22 peptides, also conferred listeriosis protection when used in dendritic cell-based vaccines. In this study, we explore GAPDH of *Listeria, Mycobacterium*, and *Streptococcus* spp. taxonomic groups to search for epitopes that confer broad protection against pathogenic strains of these bacteria. GAPDH multivalent epitopes are selected if they induce inhibitory actions and wide-ranging immune responses. Proteomic isolation of GAPDH from dendritic cells infected with *Listeria, Mycobacterium*, or *Streptococcus* confirmed similar enzymatic, Rab5a inhibitory and immune stimulation abilities. We identified by bioinformatics and functional analyses GAPDH N-terminal 1–22 peptides from *Listeria, Mycobacterium*, and *Streptococcus* that shared 95% sequence homology, enzymatic activity, and B and T cell immune domains. Sera obtained from patients or mice infected with hypervirulent pathogenic *Listeria*, *Mycobacterium*, or *Streptococcus* presented high levels of anti-GAPDH 1–22 antibodies and Th2 cytokines. Monocyte derived dendritic cells from healthy donors loaded with GAPDH 1–22 peptides from *Listeria, Mycobacterium*, or *Streptococcus* showed activation patterns that correspond to cross-immunity abilities. In summary, GAPDH 1–22 peptides appeared as putative candidates to include in multivalent dendritic based vaccine platforms for *Listeria, Mycobacterium*, or *Streptococcus*.

## Introduction

Re-emerging pathogens causing severe meningitis in adults belong to the bacterial genus Listeria, Mycobacterium, and Streptococcus. None of them are preventable bacterial pathogens at present since available bovine Calmette–Guerin (BCG) or pneumococcal vaccines are not effective for meningitis in adults. Moreover, they might cause outbreaks or recurrent infections to which the elderly is the population at the highest risk, and the involvement of the central nervous system (CNS) is a factor usually associated with a higher mortality (Pagliano et al., 2017; Marais et al., 2017). Regardless of outbreaks, as the one reported in Spain last summer, caused by Listeria monocytogenes (Herrador et al., 2019), listeriosis, tuberculosis, and pneumonia caused by Streptococcus are also opportunistic infections in adults with immunocompromised conditions as cancer patients. While research in new vaccines against bacterial pneumonia is a hot topic in European or WHO institutions and tuberculosis vaccines also receive significant efforts worldwide, this is not the case for listeriosis vaccines. In fact, listeriosis arose recently as a re-emerging infectious disease, and only experimental dendritic-based vaccines have been reported, with no development at the clinical practice (Kono et al., 2012; Calderon-Gonzalez et al., 2014; Calderon-Gonzalez et al., 2015; Torres et al., 2016). For this reason, developing vaccines that protect the adults against re-emerging bacterial infections would avoid the high mortality and morbidity they cause, as well as diminish the cost of antibiotics use in our health care systems. In this regard, vaccines for adults should consider several features related with immunosenescence that affect the vaccine responses, like the dysregulation of the innate immune system, T and B cells. The imbalance of the innate immunity implies a decrease in the functionality of antigen presenting cells, phagocytic function, and cell migration capacity of macrophages and dendritic cells (DCs) (Solana et al., 2006). T follicular helper cells required for optimal titers in T dependent vaccines appeared reduced in function and number with aging, and B cells accumulated many defects in the elderly that reduce B cell diversity, while reducing specific antibody levels and increasing the amounts of non-specific antibodies produced with aging (Weinberger, 2018).

In recent years, a new concept in vaccinology arose that can be applied in the development of vaccines for adults, cross-immunity that might support the hypothesis that multivalent vaccines protect against a broad-spectrum of bacterial pathogens. Cross-immunity implies that vaccines designed against a pathogen can confer protective immunity against different microorganisms involving innate as well as specific immunity. A putative explanation for cross-immunity regards the innate immune cells such as dendritic cells (DCs) or macrophages that act in a non-specific pattern, drive also specific immunity and serve as multivalent vaccines (Miyasaka, 2020). The search for bacterial epitopes to be included in these multivalent vaccines is currently very active. In this regard, looking for common virulence factors and shared by structural immune domains of several bacterial pathogens might help to discover vaccine candidates.

*Mycobacterium, Listeria*, or *Streptococcus spp*. belongs to unrelated taxonomic groups but shares virulence factors as well as groups of populations at high risk of infection as mentioned before. Therefore, it will be worthy to prepare multivalent vaccine designs that might confer broad protection against these three bacterial genera. In this regard, the toxins involved in host membrane disruption such as listeriolysin O (LLO) of *Listeria monocytogenes* (LM) (Nguyen et al., 2019), pneumolysin (PLY) of *Streptococcus pneumoniae* (SP) (Los et al., 2013) or mycobacteria factors of the ESX-1 secretion system such as ESAT-6, and CFP-10 of Mycobacterium tuberculosis (MTB) (Smith et al., 2008) have similar virulence factors. However, these pore-forming toxins do not share any sequence homology between them, and therefore, they hardly share immunogenic epitopes. However, the glycolytic enzyme, glyceraldehyde-3-phosphate dehydrogenase (GAPDH) of the above-mentioned bacterial pathogens can attach to cell surface immune-related proteins (*i.e.*, lactoferrin, fibrinogen, plasmin, or C1q) or intracellular GTPases involved in trafficking (Alvarez-Dominguez et al., 2008; Terrasse et al., 2012; Boradia et al., 2014; Ireton et al., 2014; Malhotra et al., 2017; Moreau et al., 2017; Myllymäki et al., 2017), contributing not only to their virulence but also to the identification of pathogen broad immunogenic epitopes to design multivalent vaccines.

## Results and Discussion

The abilities of bacterial GAPDH from LM, MTB, or SP to bind to cell surface or intracellular proteins predicted they might share binding domains that contribute to virulence and immune responses. We initiated this study with the hypothesis that GAPDH domains that contained inhibitory actions and induced the activation of innate immune cells might contain the epitopes for multivalent vaccines.

### GAPDH of *Listeria, Mycobacterium* and *Streptococcus* Are Detected in DCs and Share Binding and Immunogenic Domains

Since bioinformatic and biochemical approaches revealed that GAPDH from LM and SP contained a nicotinamide adenine dinucleotide (NAD)-binding domain with a predicted ADP ribosylation ability onto Rab5a (Alvarez-Dominguez et al., 2008), here we have extended these analyses to Mycobacterium ssp.

**Figure 1:**
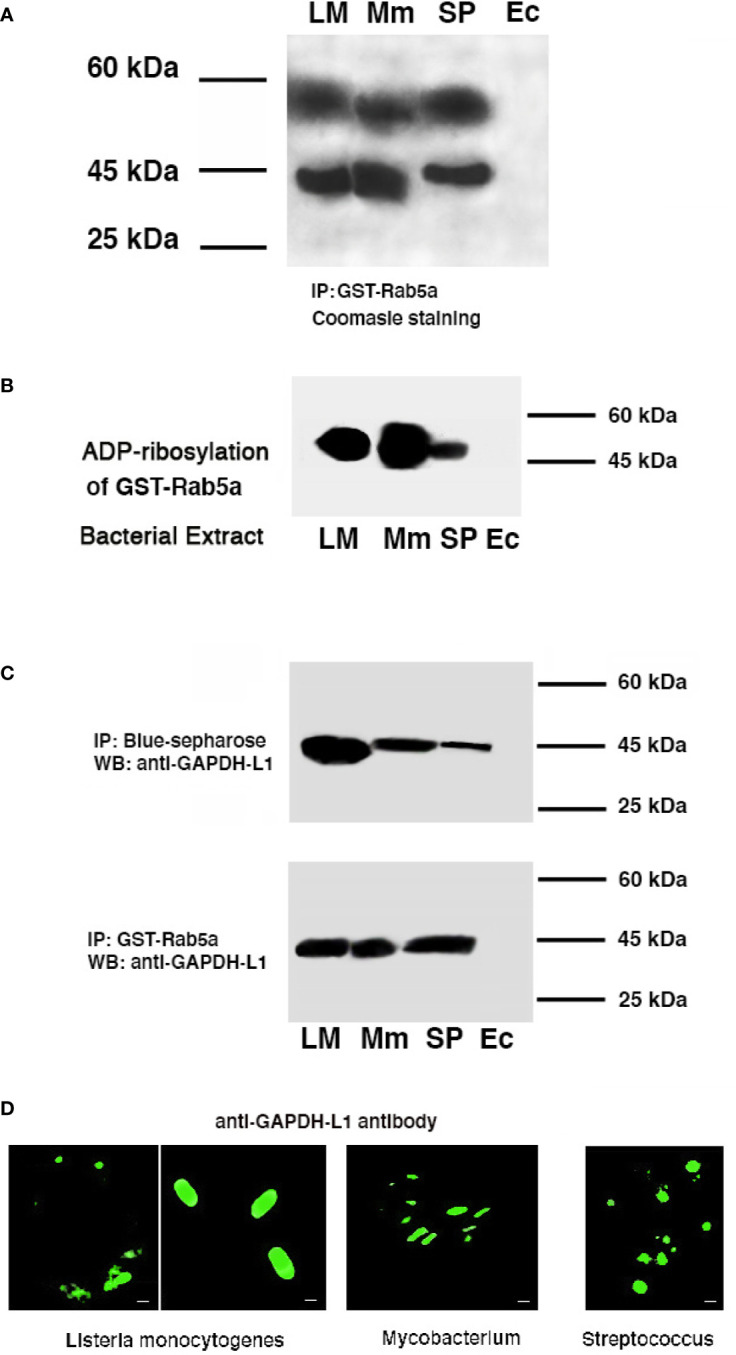
GAPDH of *Listeria, Mycobacterium*, and *Streptococcus* are detected in murine bone-marrow DC and shared binding and immunogenic domains. *Listeria monocytogenes* (LM), *Mycobacterium tuberculosis* (MTB), *Mycobacterium marinum* (Mm), *Streptococcus pneumoniae* (SP) **(A)** GAPDH isolation and proteomic characterization after DC loading with bacterial extracts of LM, MTB, or SP and immunoprecipitation of DC lysates with GST-Rab5a columns. **(B)** Recombinant Rab5a purified protein was ADP-ribosylated using NAD-biotin in the presence of different bacterial extracts, LM, MTB, SP, or *Escherichia coli* (Ec). ADP-ribosylation was performed using an ADPRT buffer in the presence of cytosolic proteins (30 µg) of bone-marrow DC. ECL was performed with streptoavidin-HRPO conjugated (1:10,000 dil). **(C)** DCs were infected with different bacterial pathogens: LM, *M. marinum* (Mm) or SP for 16 h. A set of lysates of infected DC was immunoprecipitated with Blue-sepharose to isolate NAD-binding proteins and another set immunoprecipitated with GST-Rab5a. Both immunoprecipitations were run on SDS-PAGE gels and western-blot developed with a rabbit anti-GAPDH-L1 antibody that recognized the L1 peptide. **(D)** The same DC infected as in **(C)** were fixed with p-formaldehyde, stained with rabbit anti-GAPDH-L1 and a goat anti-rabbit-FITC labeled and examined by Confocal microscopy. Scale bars correspond to 1 µm, except second *Listeria* image that correspond to 3 µm.

We loaded relevant immune cells to induce cross-immunity as DC with LM, MTB, or SP extracts (300 µg) and explored whether we could isolate bacterial GAPDH from the cells using different procedures to detect proteins sharing the same domains (**Table 1** and **Figure 1A**). In the first approach, we detected NAD-binding proteins by using Blue-sepharose NAD-affinity columns (**Table 1**, column b) (Alvarez-Dominguez et al., 2008). In the second approach, we isolated Rab5a-binding proteins using GST-Rab5a affinity columns (**Table 1**, column c). After elution from the two types of affinity columns, elutes were run on 10% SDS-PAGE gels, bands stained with Coomasie dye and cut out with sterile razors. Bands were digested with trypsin, and mass spectrometry was applied to identify the eluted proteins. As it is shown in **Figure 1A**, LM, MTB, or SP proteins eluted from Rab5a columns matched the molecular weight expected for GAPDH and were shown to correspond exclusively to GAPDH by mass spectrometry (**Table 1**, column b). However, proteins eluted from Blue-sepharose columns matched with GAPDH and enolase for LM, MTB, and SP, and two additional proteins were also eluted in the case of MTB, the heat-shock protein 70 (Hsp70), and the elongator factor 60 (EF-60) (**Table 1**, column c).

**Table 1:** Identification of eluted proteins from affinity columns of Rab5 or Blue-sepharose loaded with bacterial extracts of *Listeria, Mycobacterium*, and *Streptococcus*.

^a^Bacteria strain	^b^Proteins eluted from Rab5a column	^c^Proteins eluted from Blue-sepharose column
***Listeria monocytogenes***	GAPDH	GAPDHenolase
***Mycobacterium tuberculosis***	GAPDH	GAPDHHsp70EF-60enolase
***Streptococcus pneumoniae***	GAPDH	GAPDHenolase

^a^300 µg of DC loaded with bacterial extracts of Listeria, Mycobacterium, or Streptococcus was used to elute and identify GAPDH by different affinity columns. ^b^Proteins eluted from a Rab5a-affinity column using GST-Rab5a incubated with lysates of DC loaded with each bacterial extract. ^c^Proteins eluted from Blue-sepharose affinity columns incubated with another set of lysates of DC loaded with each bacterial extract. Elutes of a and b affinity columns were run in 10% SDS-PAGE gels and bands cut off with sterile razors, trypsin digested, and applied to mass spectrometry to identify the proteins.

We next verified that LM, MTB, and SP extracts could ADP-ribosylate Rab5a with a procedure that uses NAD-biotin (Barbieri et al., 2001; Alvarez-Dominguez et al., 2008). As it is shown if **Figure 1B**, LM, MTB, and SP extracts ADP-ribosylated Rab5a, while an *E. coli* extract did not. Therefore, GAPDH from MTB and SP shared NAD and Rab5a-binding domains and presented analogous ADP-ribosylating activities, emerging as virulence factors comparable to Lmo2459 from LM.

To validate analogous virulence and immunogenicity of GAPDH, our approach involved infection of DC with pathogenic strains of *Listeria, Mycobacterium*, and *Streptococcus* taxonomic groups (100:1 MOI) for 16 h, to apply DC lysates to Rab5a or Blue-sepharose affinity columns. Since *Mycobacterium marinum* (MM) is a human pathogen used in experimental models to screen pre-clinical MTB vaccines (Myllymäki et al., 2017), we selected this pathogenic *Mycobacterium* as it requires a more convenient level 2 of biosecurity to handle in the laboratory and animal facilities than the level 3 required by MTB. Clinical isolates of *Streptococcus pneumoniae* (SP) were used in all the experiments of this study. As it is shown in **Figure 1C**, proteins eluted from Rab5a-affinity or Blue-sepharose affinity columns of DC infected with LM, MM or, SP contained a single band of ~43 kDa molecular weight by western-blot recognized using a polyclonal anti-GAPDH-L1 antibody developed against LM. Finally, using this antibody in confocal microscopy at short times of infection, as is 1 h (**Figure 1D**, we detected LM, MM, or SP bacterial shapes in DC, indicating bacterial GAPDH was a structural protein.

In summary, GAPDH from bacterial extracts of LM (Lmo 2459), MTB, or SP or isolated from DC infected with pathogenic strains of *Listeria*, *Mycobacterium* or *Streptococcus*, shared NAD and Rab5a-binding domains and presented similar ADP-ribosylation enzymatic activities as well as immunogenic domains, revealing as a putative virulence factor to explore for multivalent domains.

### Sequence Homology and ADP-Ribosylating Abilities of Peptides 1–22 of GAPDH in the Genus *Listeria, Mycobacterium*, and *Streptococcus*

**Figure 2:**
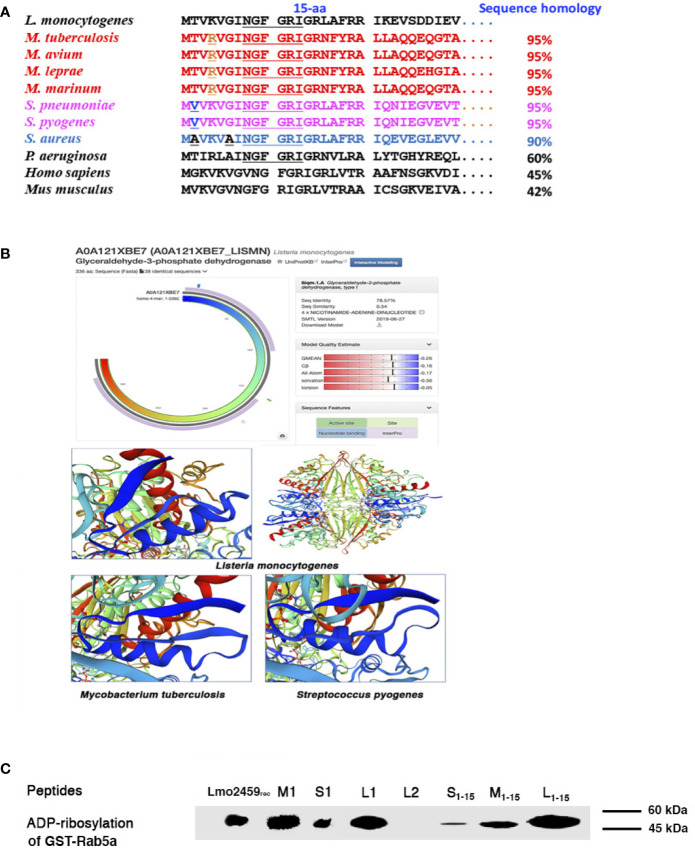
Sequence homology and ADP-ribosylating abilities of peptides 1–22 of GAPDH in the genus *Listeria, Mycobacterium*, and *Streptococcus*. **(A)** Alignments of bacterial GAPDH protein sequences of NAD-binding domains with sequence homologies higher that 90% and compared to GAPDH-Listeria. Alignments are performed using MPsrch, a comparison tool implementing the true Smith and Waterman algorithm. The protein sequences of pathogenic strains of *Mycobacterium* genus are shown in red, in pink of *Streptococcus* genus and in blue of *Staphylococcus* genus. The NAD-interacting residues are underlined in all bacteria sequences. **(B)** 3D predictions of GAPDH-*Listeria*, GAPDH-*Mycobacterium*, and GAPDH-*Streptococcus* using the SWISS-MODEL server and the available crystal structures of GAPDH-LM (A0A121XBE7_LISMN), GAPDH-MTB (A0A045ITJ4_MYCTX), and GAPDH-SP (P0C0G6, G3P_STRPY); region in blue of lower images corresponds to the first 22 amino acids of the protein and contained a ***β***-strand and an ***α***-helix structure. **(C)** ADP-ribosylation of recombinant Rab5a using peptides with different lengths such as peptides L_1–15_, M_1–15_, S_1–15_, L1, M1, S1 or L2, a negative control of GAPDH-LM 23–42 peptides. L1–15 and L1 showed the highest levels of ADP-ribosylation, while M_1–15_ and S_1–15_ show significant ADP-ribosylation abilities but slightly lower.

Next, we searched for GAPDH epitopes that served as multivalent domains in LM, MTB, and SP. As a first approach, we performed a bioinformatics analysis to search for homologies higher than 95% in the three bacterial genus, *Listeria, Streptococcus*, and *Mycobacterium* (**Figure 2A**. Other bacteria genera as *Pseudomonas* or *Staphylococcus* spp. with reported ADP-ribosylating enzymes (Barbieri et al., 2001; Myllymäki et al., 2017) presented lower GAPDH sequence homologies, varying from 60 to 85%, respectively (**Figure 2A**. In fact, sequence homology in the genera of *Listeria, Mycobacterium*, and *Streptococcus* increased to 99% in the first 15 amino acids of N-terminus. The difference between GAPDH of *Listeria* and *Streptococcus* consisted in a T residue (threonine) in position 2 of *Listeria*, aligned with a V residue (valine) in *Streptococcus*. Meanwhile, the difference between *Listeria* and *Mycobacterium* pathogenic strains was a K (lysine) residue in position 4 of *Listeria*, aligned with an R (arginine) residue in *Mycobacterium*, being K and R residues with similar cationic residues. These sequence homologies anticipated the common enzymatic activities as well as structural domains described with their bacterial extracts (**Figures 1B–D)**. SWISS-MODEL server and the available crystal structures allowed comparison of the three dimensional predictions of GAPDH-LM (A0A121XBE7_LISMN, https://swissmodel.expasy.org/repository/uniprot/A0A121XBE7), GAPDH-MTB (A0A045ITJ4_MYCTX, https://swissmodel.expasy.org/repository/uniprot/A0A045ITJ4) and GAPDH-*Streptococcus pyogenes* (GAPDH-SPY) (P0C0G6, G3P_STRPY, https://swissmodel.expasy.org/repository/uniprot/P0C0G6) (**Figure 2B** images), predicted that the NAD-interacting residues of the Rossman-fold domains, localized in the first 22 amino acids (underlined amino acids in **Figure 2B**, containing a *β*-strand (residues 3–9) and a *α*-helix with amphipathic structures (residues 11–22) (blue structures in images of **Figure 2B**. InterPro alignments of GAPDH-LM, GAPDH-MTB, and GAPDH-SPY confirmed they all presented NAD-binding domains at the N-terminus, necessary for ADP-ribosylation, and G3P-dehidrogenase catalytic domains at the C-terminus **(Supplemental Figure S1A**). In fact, we verified with highly purified 1–22 peptides of GAPDH-LM (L1), GAPDH-MTB (M1), or GAPDH-SP (S1), the ADP ribosylated Rab5a, as previously reported for L1 peptide (Alvarez-Dominguez et al., 2008) and comparable to ADP-ribosylating levels of recombinant Lmo2459 (**Figure 2C)**. We limited the minimal enzymatic activity of peptide 1–22 of GAPDH-LM, GAPDH-MTB, and GAPDH-SP to a shorter peptide 1–15 with higher 99% sequence homology (**Figure 2A)**, that ADP-ribosylated Rab5a similarly to longer 1–22 peptides L1, M1, and S1 peptides (L_1–15_, M_1–15_ and S_1–15_ bands in **Figure 2C**). However, peptide 23–42 of GAPDH-LM (L2) containing the G3P-dehydrogenase catalytic domain, showed no ADP-ribosylating abilities as expected (**Figure 2C**). We concluded that GAPDH-LM, GAPDH-MTB, and GAPDH-SP contained analogous 1–15 and 1–22 epitopes, including both the enzymatic and protein binding activities.

### Immune Responses Elicited by Peptides of GAPDH-LM, GAPDH-MTB, and GAPDH-SP

**Figure 3:**
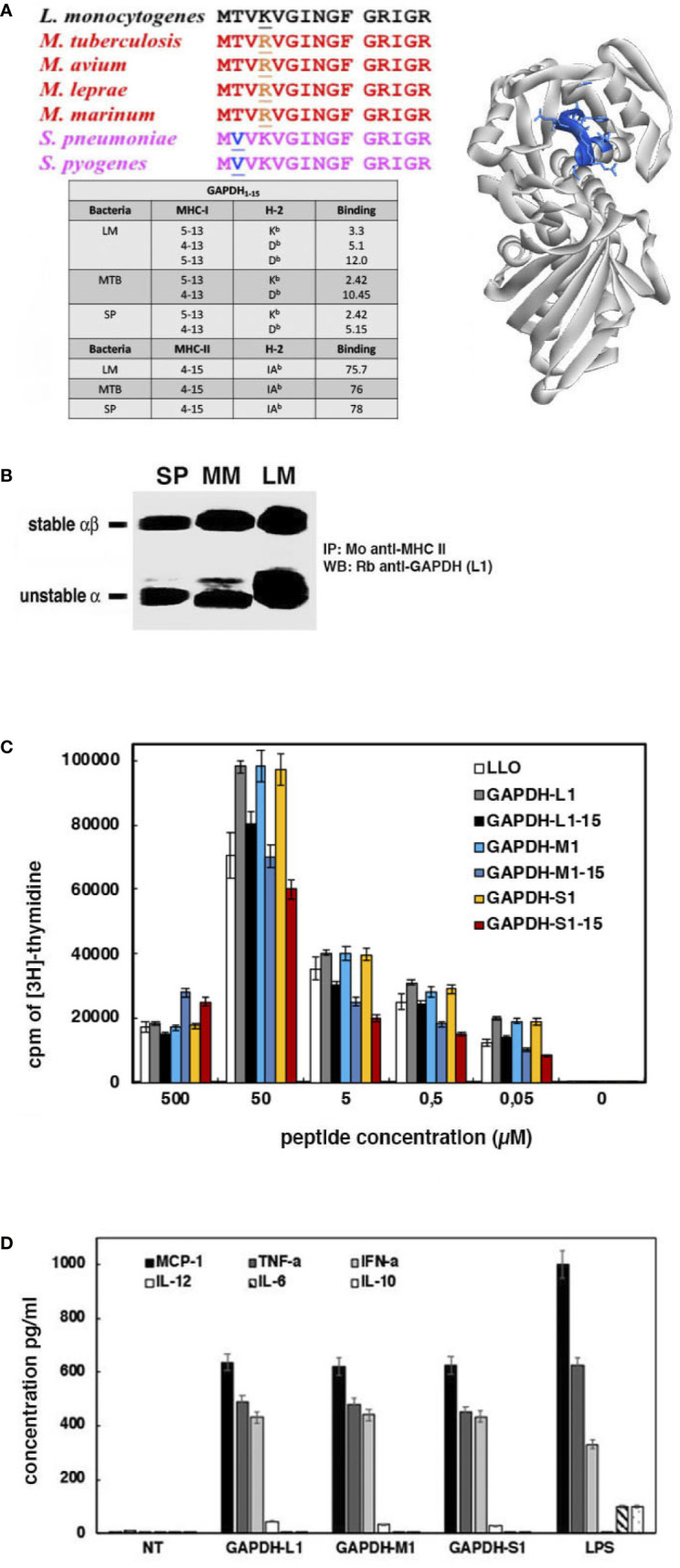
Immune responses elicited by L1, M1 and S1 peptides of GAPDH and clinical significance of their use as biomarker tools. **(A)** Alignments of bacterial GAPDH protein sequences of NAD-binding domains with sequence homologies higher than 99% and compared to GAPDH-LM (upper sequence in black). Underlined are the residues that differ from the GAPDH-LM sequence. On the right image is the predicted 3D structure of GAPDH-LM showing the 4–15 residues in blue that contained the MHC binding epitopes. The lower image corresponds to a table compiling MHC predictions performed with IEDB Consensus tool, indicating the binding epitopes to MHC class I and II molecules. **(B)** Lysates of DC infected with LM, MM or SP as in **Figure 2C** were immunoprecipitated with monoclonal anti-MHC-IA^b^ (clone Y3P); immunoprecipitates were run on SDS-PAGE gels and western blots developed with anti-GAPDH-L1 antibody. The MHC-II stable and unstable forms in SDS-PAGE are shown as markers. **(C)** DC loaded with the different peptides, L1, L1–15, M1, M1–15, S1, M1–15 were inoculated into the right hind footpads of mice. Popliteal nodes were collected, homogenated, and cultured ***in vitro*** in the presence of each corresponding peptide. Plot shows the T cell proliferation after [^3^H]-thymidine incorporation. **(D)** MoDCs from healthy donors were incubated with L1, M1, or S1 peptides for 16** h**, and filtered supernatants were examined for cytokine levels (pg/ml).

To predict the minimal epitope requirements to prepare a DC vaccine that elicited T cell responses, we restricted our analysis to the 1–15 peptide of GAPDH sequences from LM, MTB, and SP as they shared 99% sequence homology (**Figure 3A**). Next, we used the IEDB Consensus tool bioinformatics approach to predict GAPDH binding to MHC molecules. IEDB analysis envisaged that percentile ranks <10 corresponded to good MHC class I binders, while percentile ranks <100 are weak binders. In this regard, percentile thresholds <50 correspond to good MHC class II binders, while thresholds <500 are intermediate binders (Nielsen et al., 2003; Peters and Sette, 2005; Sidney et al., 2008; Lundegaard et al., 2008; Kim et al., 2012; Calderon-Gonzalez et al., 2015; Rack et al., 2015; Stephenson et al., 2015; Andreatta and Nielsen, 2016). In this regard, GAPDH 1–15 sequences contained two epitopes predicted as good binders for K^b^ and D^b^ MHC class I molecules, 5–13 and 4–13 amino acids, respectively, and one epitope predicted as intermediate binder for IA^b^ MHC class II molecules, 4–15 amino acids (table with peptide MHC binding sequences in **Figure 3A**). Moreover, these GAPDH 4–15 epitopes and predicted binders for MHC molecules are similar in LM, MTB, and SP sequences. The theoretical 3D model revealed that GAPDH 4–15 epitopes included one tight loop and one *α*-helix, 3D structures characteristic of MHC class I and II epitopes, respectively (image on the right showing 4–15 amino acids in blue, **Figure 3A**). We verified these binding predictions in DC infected with either LM, MM, or SP after immunoprecipitation of MHC class II molecules with a monoclonal anti-MHC class II antibody (clone Y3P that recognized IA^b^ in C57BL/6 mice) and western-blot analysis with anti-GAPDH-L1 antibody (**Figure 3B**). GAPDH 1–22 peptides from LM, MM, and SP were detected on stable and unstable forms of immunoprecipitated MHC class II molecules, indicating that *in vivo* generated GAPDH immunogenic epitopes are capable to bind to MHC molecules and predict GAPDH antigen presentation and induction of immune responses.

In fact, we confirmed that GAPDH 1–15 and 1–22 peptides from LM, MM, and SP elicited efficient T cell responses after immunizing mice with LM, MM, or SP (*model in* **Supplemental Figure S1B** *, approach 1*). Next, we loaded DC with the following peptides from LM, MM or SP sequences of GAPDH: L1, L_1–15_, M1, M_1–15_, S1 or S_1–15_. Peptide loaded DCs were inoculated into the hind foot pads of C57BL/6 mice (10^6^ cells/foot pad) together with adjuvant DIO-1 (2 µg/ml) to collect popliteal lymph nodes. DIO-1 is an adjuvant that binds to TLR2/4 molecules (Guisasola and Escudero, 2016). We re-stimulated homogenates of popliteal lymph nodes *in vitro* with different concentrations of peptides, from 0.05 to 50 µM (L1, L_1–15_, M1, M_1–15_, S1 or S_1–15_) and examined immune responses by classical proliferation assays of lymphocytes using [^3^H]-thymidine. We also included in the assay recombinant listeriolysin O (LLO) (3 µg/hind foot pad) as a positive control. As it is shown in **Figure 3C**, peptides L_1–15_, M_1–15_, and S_1–15_ elicited T cell responses similar to LLO, but lower than T cell responses of 1–22 peptides (L1, M1 or S1). We concluded that both 1–22 and 1–15 peptides elicited T cell immune responses in the same range or higher than the highly immunogenic bacterial protein, LLO.

### Significance of Two Biomarkers as Tools to Design Clinical Multivalent Vaccines Against Listeriosis, Tuberculosis, and Pneumonia Caused by *Streptococcus pneumoniae*

To verify the significance of our hypothesis that GAPDH was a virulence factor common to LM, MTB, and SP infections that presented multivalent epitopes, we collected sera and clinical isolates of patients with listeriosis, tuberculosis, or pneumonia caused by SP, selected from a 2014–2018 study in our institution (Department of Microbiology, HUMV) (patients with asterisks in **Supplemental Table S1**). Sera of patients infected with hypervirulent strains of listeriosis, tuberculosis or *Streptococcus* pneumonia presented high levels of anti-GAPDH-L1 antibodies, OD ≥ 2.0 (**Table 2**, column c). We also checked for cytokines in sera of patients and detected threefold higher levels of IL-6 and IL-10, classical Th2 cytokines compared to controls (**Table 2**, column d).

**Table 2:** Clinical data of patients infected with hypervirulent LM, MTB, and SP.

^a^Strain code(PATIENTS)	^c^Anti-GAPDH-L1 antibodies	^d^CYTOKINES		
		IFN	IL-6	IL-10
**^a^HUMV-LM01**	2.4 ± 0.1	5 ± 0.2	10 ± 0.1	6 ± 0.1
**HUMV-MTB01**	2.2 ± 0.2	4 ± 0.1	7 ± 0.1	5 ± 0.1
**HUMV-SP01**	1.9 ± 0.1	4 ± 0.2	9 ± 0.1	5 ± 0.1
**^b^NI-control**	0.13 ± 0.1	2 ± 0.1	3 ± 0.1	2 ± 0.1

^a^Clinical isolates from patients at HUMV (Microbiology Dpt.) selected from a 2014 to 2019 study as hypervirulent strains (complete study in Supplemental file). ^b^Control data correspond to adult healthy donors at HUMV and non-infected. ^c^Sera from patients selected (HUMV-LM01, HUMV-MTB01, and HUMV-SP01) were examined for anti-GAPDH-L1 antibodies by a peptide ELISA. Results are presented as the mean ± SD of OD units in triplicate measurements (P < 0.05). ^d^Cytokine concentrations were analyzed in sera of patients by flow cytometry (pg/ml). Results are expressed as the mean ± SD concentrations (pg/ml) of three different experiments. ANOVA was applied to flow cytometry results and a Student’s t test to ELISA analysis.

Next, we confirmed the hypervirulence of selected clinical isolates of LM, MM, or SP after inoculation of C57BL/6 mice intravenously (*i.v*) with 10^4^ CFU (**Table 3**). Fourteen days later, we recovered sera and spleens and examined the levels of anti-GAPDH-L1 antibodies using the ELISA-peptide previously described (Calderon-Gonzalez et al., 2016a; Calderon-Gonzalez et al., 2016b) (*model shown in* **Supplemental Figure S1B** *, approach 1*). Several non-pathogenic strains of each pathogen were also included as controls, a listeriolysin deficient mutant of LM (LM-ΔLLO), a non-pathogenic strain of mycobacteria (*M. smegmatis*), and a vaccine strain of SP (ATCC 49619-19F). All non-pathogenic strains presented low levels of anti-GAPDH-L1 antibodies (OD ≤ 0.5) (**Table 3**, column d). Also, non-pathogenic bacteria did not induce higher levels of Th1 or Th2 cytokines (**Table 3**, column e), while sera of mice infected with hypervirulent strains presented high levels of Th2 cytokines, IL-6 and IL-10. Hypervirulent clinical isolates of LM, MTB, or SP showed at least 100-fold higher CFU numbers than non-pathogenic bacterial strains (**Table 3**, column f) and high levels of anti-GAPDH-L1 antibodies (OD ≥ 0.65) (**Table 3**, column d). These results validated the correlation between the levels of anti-GAPDH-L1 antibodies and bacterial virulence. We repeated all these ELISA-peptide experiments for detection of antibodies to peptides M1 and S1 and confirmed all hypervirulent strains produced high levels of anti-GAPDH antibodies (data not shown). We also confirmed specific T cell responses to L1 peptide in spleen homogenates of mice, evaluating the percentages of CD4^+^ and CD8^+^ cells stimulated with L1 peptide. As it is shown in **Table 3** (column g), spleen homogenates of mice inoculated with pathogenic bacteria and next stimulated *in vitro* with L1 peptide showed percentages of T cells in ranges of 14% for CD4^+^ and 17% for CD8^+^ positive cells that were much larger than percentages detected in controls with less virulent or non-pathogenic strains, 8% for CD4^+^ T cells or 3% for CD8^+^ T cells. These results revealed that titers of anti-L1 (anti-M1 and anti-S1) antibodies were valid biomarkers to detect high immune responders among patients with listeriosis (Calderon-Gonzalez et al., 2017), tuberculosis, or pneumonia caused by SP and therefore, valid epitopes to incorporate into multivalent vaccines.

**Table 3:** Immune parameters of mice infected with hypervirulent LM, MTB and SP.

^a^Strain code(MICE)	^d^Anti-GAPDH-L1 antibodies	^e^CYTOKINES			^F^virulence (CFU/ml)	^g^CD4+%	^g^CD8+%
		IFN	IL-6	IL-10			
**^a^HUMV-LM01**	2.85 ± 0.1	4 ± 0-1	9 ± 0.1	5 ± 0.1	2.9 × 10^5^ ± 10	12 ± 0.2	17 ± 0.3
**HUMV-MTB01**	2.20 ± 0.2	4 ± 0.2	8 ± 0.1	4 ± 0.1	3.9 × 10^4^ ± 10	9.5 ± 0.8	14 ± 0.3
**HUMV-SP01**	1.81 ± 0.1	3 ± 0.1	9 ± 0.1	5 ± 0.1	3.8 × 10^5^ ± 13	10 ± 0.7	13 ± 0.3
**^b^LM^ΔLLO^ mutants*M. smegmatisS. pneumoniae*^49619-19F^**	0.42 ± 0.10.75 ± 0.10.67 ± 0.2	2 ± 0.11.2 ± 0.11± 0.1	3± 0.12.5± 0.12.8 ± 0.1	2 ± 0.12.1 ± 0.12.0 ± 0.1	4.2 × 10^0^ ± 101.5 × 10^2^ ± 81.2 × 10^3^ ± 10	8 ± 0.19 ± 0.19 ± 0.2	1 ± 0.11 ± 0.11 ± 0.1
**^c^NI-control**	0.12 ± 0.1	2 ± 0.1	3 ± 0.1	2 ± 0.1	0.1 × 10^0^ ± 0.1	2 ± 0.2	1.5 ± 0.1

^a^Hypervirulent strains of LM, MM or SP as in **Table 1** and ^b^non-virulent strains as LLO deficient LM mutant, LM^ΔLLO^, M. smegmatis or the SP vaccine strain S. pneumoniae^49619-19F^ were used to i.v. inoculate female C57BL/6 mice (n = 5) with 5 × 10^3^ CFU bacteria. 14 days later, mice were bled, sacrificed, and spleens collected. ^c^Control corresponds to non-infected (NI) mice. ^d^Mice sera were examined for anti-GAPDH-L1 antibodies by a peptide ELISA. Results are presented as the mean ± SD of OD units in triplicate measurements (P < 0.05). ^e^Cytokine concentrations were also analyzed in mice sera by flow cytometry (pg/ml). Results are expressed as the mean ± SD concentrations (pg/ml) of three different experiments. ^f^Virulence of different bacteria was measured in homogenized spleens (1 ml) after plating in blood agar plates. CFUs were counted and results expressed as CFU/ml (P ≤ 0.5). ^g^Aliquots (100 µl) of the spleen homogenates were analyzed for CD4^+^ and CD8^+^ cell populations by FACS. Results are expressed as percentages of positive cells. ANOVA was applied to all flow cytometry results. Student’s t test was applied to ELISA and virulence assays.

The other parameters of interest to design multivalent vaccines correspond with the ability of epitopes to induced cross-immunity (Miyasaka, 2020). To search for cross-immune epitopes in GAPDH from LM, MTB and SP, we selected monocyte derived DC (MoDC) of healthy donors as DCs are relevant antigen presenting cells to drive cross-immunity. Next, we performed a classical DC activation analysis that checked the activation markers and cytokine production of MoDC as described (Calderon-Gonzalez et al., 2016a; Calderon-Gonzalez et al., 2017) (*model described in* **Supplemental Figure S1B** *, approach 2*). Cross immune epitopes might activate DC to produce Th1 cytokine patterns (Miyasaka, 2020). We used LPS (10 ng/ml) as a positive control of general activation of DC, inducing Th1 and Th2 cytokines. After 16 h of MoDC incubation with peptides, L1, M1, or S1, we explored the cell surface markers and cytokine production in supernatants. Cell surface markers confirmed that MoDC treated with L1, M1, or S1 peptides presented the following phenotype: 90% of CD45^+^HLA-DR^+^CD80^+^CD86^+^CD14^−^ positive cells, while LPS treated MoDC presented 90% of CD45^+^HLA-DR^+^CD80^+/−^CD86^+/−^CD14^−^ positive cells. These results indicated that these GAPDH peptides induced a MoDC activated phenotype. Cytokine analysis in MoDC supernatants detected high levels of MCP-1, TNF-α, IFN-α, and IL-12 after incubation with L1, M1, or S1 peptides, a classical Th1 cytokine pattern (**Figure 3D**). Incubation with LPS also induced Th2 cytokines, IL-6, and IL-10. These results revealed that induction of Th1 cytokines by MoDC was a valid biomarker to detect bacterial epitopes that induced TI.

## Conclusions

GAPDH from LM, MTB, and SP emerged as a common virulence factor that contained 1–15 and 1–22 amino acid epitopes able to act with analogous enzymatic activities, MHC binding properties and induced B and T cell immune responses, with broad and specific capacities. These GAPDH 1–22 epitopes (L1, M1, or S1 peptides) induced Th1 activation of DC, a relevant biomarker of bacterial TI-epitopes. Moreover, antibody titers against GAPDH 1–22 epitopes in patients with listeriosis, tuberculosis, or pneumonia are good biomarkers to select patients infected with hypervirulent strains but high T cell responders, putative candidates to explore the efficiency of DC or multivalent vaccines.

Vaccination is the best tool to prevent infections with re-emerging pathogens as *Listeria monocytogenes, Mycobacterium tuberculosis*, or *Streptococcus pneumoniae*, that together with respiratory virus as Influenza or SARS or SARS-COV-2 coronavirus, cause severe infections in the elderly and adults with immunosuppressive conditions. Therefore, deciphering epitopes with broad but specific action as well as broad virulence activity appears as the first approach for DC and multivalent vaccine designs.

## Data Availability Statement

The raw data supporting the conclusions of this article will be made available by the authors, without undue reservation.

## Ethics Statement

The studies involving human participants were reviewed and approved by Ethical Committee of Clinical Research of Cantabria at Instituto de Investigación Marqués de Valdecilla (Santander, Spain), with the reference number 2014.228. The patients/participants provided their written informed consent to participate in this study. The animal study was reviewed and approved by Ethical Committee of Animal Experiments of the University of Cantabria (Permit Number: PI-01-17).

## Author Contributions

CA-D, DS-C, HT-N, and RC-G performed the experiments. RT performed the bioinformatic binding predictions. IG and SG helped with the chemical ADP-ribosylating assays and bioinformatic analyses. AP performed the proteomics assays of gel bands and the identification using mass spectrometry. AS provided *M. smegmatis.* FJS and AS helped with the mycobacteria assays as they are experts on mycobacteria handling in mice. MF synthesized the DOI-1 adjuvant, helped with the immune assays, provide funding for several and experiments, and help to design the hypothesis of the study. JC-M and CP provided with the bacterial clinical isolates from patients with listeriosis, tuberculosis and SP pneumonia and helped with the bacterial analyses. SY-D provided with *M. marinum* clinical isolate from patients. CA-D directed the study and designed the experiments. All authors contributed to the article and approved the submitted version.

## Funding

This research was funded by the CICYT program of the Ministry of Science and Innovation, grant number SAF2012-34203, the Instituto de Salud Carlos III (ISCIII) grant numbers, DTS18-00022 and PI19-01580 to CA-D, the intramural CIBER-BNN grant number, CIB16-NM009 to IG and CA-D, to the Bio-health Research Program of Cantabria Government, grant number INNVAL19/26 to SY-D, CM, and CA-D; SAF2016-75988-R “Comunidad de Madrid (S2017/BMD-3671. INFLAMUNE-CM) and Fondo de Investigaciones Sanitarias” (BIOIMID) to MF and a Predoctoral contract to DS-C by Bio-health research program of Cantabria government. This study was co-funded in part with European FEDER funds, "A new way of making Europe" as well as funds from the COST European action ENOVA.

## Conflict of Interest

This study is protected by the following patent PCT/ES2019/070413, entitled to the Instituto de Investigación Marqués de Valdecilla.

The authors declare that the research was conducted in the absence of any commercial or financial relationships that could be construed as a potential conflict of interest.
