# Supplementary material for: Epitopes for Multivalent Vaccines Against Listeria, Mycobacterium and Streptococcus spp: A Novel Role for Glyceraldehyde-3-Phosphate Dehydrogenase
Source: Front Cell Infect Microbiol. 2020 Oct 28;10:573348. doi: 10.3389/fcimb.2020.573348 (PMC7657268; doi:10.3389/fcimb.2020.573348)
Supplement: Supplementary file 1 [file DataSheet_1.docx]

Supplementary Material

# Supplementary Materials and Methods.

## Mice. We used C57BL/6 mice from our animal facilities at the University of Cantabria at 20–24 weeks old, an age that mimics in human beings 50-years of age and older.

## Bacteria, peptide, adjuvants and cells. We used *L. monocytogenes* strains 10403S (LM^WT^) and LM^∆LLO^ mutants derived from 10403S strain (D.A. Portnoy, Berkley University, CA, USA). *M. smegmatis* strain belongs to the collection of the Microbiology Department at Universidad de Cantabria (Santander, Spain), *M. marinum and S. pneumoniae* were clinical isolates of the Microbiology Department at our institution (Hospital Universitario Marqués de Valdecilla, Santander, Spain). LLO_91–99_, GAPDH_1–22_ (L1), GAPDH_1-15_ (L_1-15_) and GAPDH_23-42_ (L2) peptides of *L. monocytogenes*; GAPDH_1-22_ (M1) and GAPDH_1-15_ (M_1-15_) peptides of *M. tuberculosis* and GAPDH_1-22_ (S1) and GAPDH_1-15_ (S_1-15_) peptides of *S. pneumoniae,* were synthesized at Centro Nacional de Biotecnología (CSIC, Madrid, Spain) followed by HPLC and mass spectrometry using a MALDI-TOF Reflex IV spectrometer. Peptide purity was ≥95% after HPLC. DIO-1 is a TLR2/4 targeted molecule that we used as adjuvant [30]. Bone-marrow derived dendritic cells (DC) were cultured in DMEM complete medium as above but with 10% FCS, grew up to 10^10^ cells/mL and homogenized on Hepes-saline-EDTA buffer to obtain cytosol for ADP-ribosylation assays. ADP-ribosylation was performed as previously reported [14]. Bone-marrow-derived macrophages (BMDMs) or bone-marrow-derived dendritic cells (DCs) were obtained from femurs of 8–12-week-old female mice. BMDMs or DCs were cultured at 2 × 10^6^ cells/ml in six-well plates in Dulbecco’s Modified Eagle’s Medium (DMEM) supplemented with 20% foetal calf serum (FCS), 1 mM glutamine, 1 mM nonessential amino acids, 50 µg/ml gentamicin and 30 µg/ml vancomycin (DMEM complete medium) and 25 ng/ml macrophage colony-stimulating factor (M-CSF) for BMDMs or 25 ng/ml granulocyte–macrophage colony-stimulating factor (GM-CSF). On Day 7, the cells were harvested and analysed by fluorescence-activated cell sorting (FACS) to evaluate cell surface markers and appropriate differentiation of BMDMs or DCs using the following markers: CD11b–fluorescein isothiocyanate (FITC), CD11c–phycoerythrin (PE), IA^b^–APC, F4/80–PE, CD80–FITC, and CD86–V450. Cells were collected using cell scrapers for detaching adherent cells. In certain samples we also used after detaching adherent cells positive selection using anti-mouse CD11c-coated magnetic beads and MACS^TM^ separation columns (Miltenyi Biotech Inc., Auburn, CA) on day 7 as previously described (4).

## Bioinformatics analyses. Bioinformatics analyses. GAPDH-LM similarity searches were done via Internet using FASTA available at (http://www.ebi.ac.uk/fasta33/) and BLAST available at (<http://www.ebi.ac.uk/blast2/>) and (http://www.ncbi.nlm.nih.gov/sutils/genom_table.cgi). The analysis of protein domains was based on Pfam database available at: (http://www.sanger.ac.uk/Software/Pfam/). The theoretical 3D predictive models for *Listeria monocytogenes* GAPDH (GAPDH-LM), *M. tuberculosis* GAPDH (GAPDH-MTB) and *Streptococcus pyogenes* GAPDH (GAPDH-SP) were produced using the Automated Comparative Protein Modelling Server SWISSMODEL available at: (http://www.expasy.ch/swissmod/SWISSMODEL.html). Sequences of GAPDH-LM, GAPDH-MTB and GAPDH-SP were aligned using MPsrch, a comparison tool implementing the true Smith and Waterman algorithm available at: http://www.ebi.ac.uk/MPsrch/.

## Recombinant proteins and ADP-ribosylation. Recombinant proteins and ADP-ribosylation. Recombinant, full-length LLO cDNA (LLOrec) was expressed in large quantities as His-fusion protein in *E. coli* strain BL21 upon induction with 1 mM IPTG for 5 h at 37°C. His-tagged recombinant proteins were purified with TALON resin, according to the manufacturer’s instructions (Clontech). Recombinant GAPDH of *L. monocytogenes* (GAPDHrec), pneumolysin O (PLY) of *S. pyogenes* (PLYrec) and Ag85A of *M. marinum* (Ag85Arec) were obtained as cDNAs and expressed in large quantities as His-fusion protein in *E. coli* strain BL21 as above. For ADP-ribosylation of recombinant Rab5a, 3 µg of protein was incubated with 50 µM NAD-biotin at 37ºC in buffer 2X ADPRT (Tris-ClH 50 mM, pH 7.6, 10 mM ATP, 200 mM MgCl2, 20 mM NAD, 2 mM ADP-ribose) according with a method previously described (Zhang, 1997) and including 30 µg of a cytosolic extract of J-7774 cells, a source of ADP-ribosylating co-factors and in the presence of the following sources: 3 µg of bacterial extracts, purified GAPDH-LM, different GAPDH_1-22_ peptides (L1, M1, S1 or L2) or different GAPDH_1-15_ peptides (L_1-15_, M_1-15_, S_1-15_).

## Protein digestion. Individual SDS-PAGE gel bands were deposited in 96-well plates and processed automatically in a Proteineer DP (Bruker Daltonics, Bremen, Germany). The digestion protocol used was described by Shevchenko *et al.* [A. Shevchenko, M. Wilm, O. Vorm, M. Mann, Anal Chem 68 (1996) 850] with minor variations. After digestion, tryptic peptides were dried by speed-vacuum centrifugation and resuspended in mobile phase A (0.1% formic acid in water) for LC ESI-MSMS.

## LC ESI-MSMS analysis. NanoLC ESI-MSMS analysis was performed using an Ultimate 3000 nanoHPLC (Dionex, Sunnyvale, California) coupled to an HCT Ultra ion-trap mass spectrometer (Bruker Daltonics, Bremen, Germany). The analytical column was a reversed phase column C18 PepMap 75 µm × 15 cm, 3 µm particle size and 100 Å pore size (Dionex, Sunnyvale, California). The trap column was a C18 PepMap (Dionex, Sunnyvale, California), 5 µm particle diameter, 100 Å pore size, switched on-line with the analytical column. The loading pump delivered a solution of 0.1% trifluoroacetic acid in 98% water / 2% acetonitrile (LabScan, Gliwice, Poland) at 30 µL/min. The nanopump provided a flow-rate of 300 nL/min and was operated under gradient elution conditions, using 0.1% formic acid (Fluka, Buchs, Switzerland) in water as mobile phase A, and 0.1% formic acid in 80% acetonitrile / 20% water as mobile phase B. Gradient length was 48 min and injection volume was 5 µL. The LC system was coupled via a nanospray source (Bruker Daltonics, Bremen, Germany) to a 3D ion trap mass spectrometer operating in positive ion mode with the capillary voltage set at 1400 V. Automatic data-dependent acquisition allowed to obtain sequentially both full scan (m/z 350-1500) MS spectra followed by tandem MS CID spectra of the four most abundant ions. Dynamic exclusion was applied to prevent the same m/z from being isolated for 1 min after its fragmentation.

## Protein identification. MS and MS/MS data obtained were processed using DataAnalysis 3.4 (Bruker Daltonics, Bremen, Germany). For protein identification, MSMS spectra (in the form of mascot generic files) were searched against UniProtKB (<http://www.uniprot.org>) or NCBInr (<http://www.ncbi.nlm.nih.gov/>). Database searches were done using Mascot v.2.2.04 ([www.matrixscience.com](http://www.matrixscience.com); Matrix Science, London, UK). Search parameters were set as follows: carbamidomethyl cysteine as fixed modification and oxidized methionine as variable one. Peptide mass tolerance was set at 0.6 Da both in MS and MS/MS mode, and 1 missed cleavage was allowed. In most cases, an accuracy of ± 0.2-0.3 Da was found both for MS and MS/MS spectra. FDR ≤ 5% for peptide identification were manually assessed as follows: after database searching, a set of peptide matches was ranked according to their corresponding Mascot scores. This list contains peptide sequences matching either forward or reversed database sequences. Finally, a subset containing 5% of peptides matching the reversed sequences was extracted.

## Isolation of MoDC, differentiation and activation. Monocytes (Mo) from healthy donors were first isolated as leukocytes from a Ficoll gradient of whole blood cells (EDTA-containers). Leukocytes recovered from the interphase were washed twice in Hank´s buffered solution and prepared in MACS^TM^ buffer (PBS-0.5% BSA-2 mM EDTA) with microbeads conjugated to mouse IgG2a monoclonal anti-human CD14 antibody (Miltenyi). CD14^+^ positive cells were selected using MACS^TM^ columns (Miltenyi, Bergisch Gladbach, Germany). FACS analysis following CD14^+^-MACS^TM^ selection indicated 99% of CD45^+^CD14^+^ cells positive cells. MoCD14^+^ cells were differentiated to MoDC at 1 x 10^6^ cells/ml in 6-well plates (Falcon^TM^) during 7 days using GM-CSF (50 ng/ml) and IL-4 (20 ng/ml) in RPMI-20%FCS medium. All differentiated cells were 98% CD45+DR+/-CD86-CD14- positive cells using specific monoclonal antibodies (Miltenyi, Bergisch Gladbach, Germany). These cells were used for *in vitro* virulence analysis as well as for activation analysis. MoDC were activated with 50 μg/mL of different LM, MTB or SP peptides (GAPDH-L1, GAPDH-M1 or GAPDH-S1) prepared in RPMI-20% FCS medium for 16 h, including as positive control LPS (10 ng/mL). Next, we checked the cell surface phenotype and detected that all cells treated with peptides presented the following phenotype: 90% of CD45^+^HLA-DR^+^CD80^+^CD86^+^CD14^-^ positive cells, while LPS treated MoDC presented 90% of CD45^+^HLA-DR^+^CD80^+/-^CD86^+/-^CD14^-^ positive cells. Supernatants of MoDC were filtered and stored at -80ºC to measure cytokines.

## Preparation of GAPDH peptide pulsed DC in mice. Bone-marrow derived DC cells obtained from femur bone-marrow, differentiated with GM-CSF (20 ng/mL) for 7 days. Next, cultured DCs were loaded with 50 μg/ml of GAPDH-L1, GAPDH-M1, GAPDH-S1, GAPDH-L1–15, GAPDH-M1-15 or GAPDH-S1-15 for 16 hours.

## T cell responses elicited by DC-GAPDH peptides. For delayed type hypersensitivity (DTH) analysis, C57BL/6 mice were immunized *i.p* with LM, MM or SP (5 x 10^3^ CFU). Seven days later, mice were inoculated in left hind footpads with DC vaccines (10^6^ cells/mice) in the presence of DIO-1 (2 μg/mL). Non-inoculated right hind footpads served as negative controls. After 48 h, we measured the footpad thickness with a caliper. T cell results in the footpads are expressed in millimeters as the mean of three different experiments ± SD. Four days later, poplyteal nodes were isolated from right hind footpads, homogenated and cultured *in vitro* with GAPDH peptides (see *Figure S1B, approach 2*).

## *In vivo* virulence of clinical isolates obtained from patients with LM, MTB or SP. C57BL/6 female mice were inoculated intravenously (i.v) with 10^4^ CFU of the following clinical isolate: HUMV-LM01, HUMV-MTB01, HUMV-SP01 or non-virulent bacteria, LM^∆LLO^ bacterial mutant, *M. smegmatis, S. pneumoniae* ATCC vaccine strain 49619-19F. 72 hours post-inoculation, mice were sacrificed and spleens recovered, homogenized and viable (CFU) bacteria examined in agar plates. Results are expressed as the mean of CFU ± SD. All data were performed in triplicate and we performed three independent experiments.

## Immunoprecipitation of MHC-II molecules in DC. Bone-marrow derived DC (DC) mice were infected with LM, MM or SP for 16 hours. Cell lysates were immunoprecipitated with mouse anti-IAb antibody (Y3P). Immunoprecipitates were run onto SDS-PAGE, transferred to nitrocellulose membranes and incubated with primary antibody, rabbit anti-GAPDH-L1 and horseradish- peroxidase-conjugated secondary antibodies.

## FACS analysis. Cell surface markers of MoDC or murine spleens were analyzed by FACS using the following antibodies: anti-DR-FITC, anti-CD45-PerCP, anti-CD86-brilliant blue and anti-CD14-PE, anti-CD4-APC, anti-CD8-FITC (Miltenyi, Bergisch Gladbach, Germany). Cytokines in patients or donor’s sera and MoDC supernatants were quantified using the CBA kit (BD Biosciences, San Jose, CA, USA). IL-17A/IL-6 ratios are expressed as the mean of ratio units (U) of triplicates ± SD. ANOVA was applied to these samples according to manufacture´s instructions. Data were analysed using the FlowJo software.

## ELISA to measure GAPDH-L1, GAPDH-M1 and GAPDH-S1 titers. GAPDH-L1, GAPDH-M1, GAPDH-S1 peptides (50 μg/ml) were coated to 96-well plates in carbonate buffer (pH 8.0) overnight at 4ºC. Plates were washed and incubated with 1 mg/ml of BSA (fraction V) to saturate all sites in the plates. Sera of listeriosis, tuberculosis or pneumonia patients or mice models of infections were 1/10 diluted and peptide coated plates incubated with diluted sera for 2 hours at RT as described (6, 27). Reactions were developed with goat anti-human IgG or goat anti-mouse IgG and absorbance analysed at 450 nm. Results are presented as optical units (OD) and mean values ± SD of triplicate experiments.

## Statistical analysis. For statistical analysis, Student's t test was applied. ANOVA analysis was applied to cytokine measurements. P ≤ 0.05 was considered significant. GraphPad software was used for generation of graphs.

## Ethics statement. This study was carried out in accordance with the Guide for the Care and Use of Laboratory Animals of the Spanish Ministry of Science, Research and Innovation. The Committee on the Ethics of Animal Experiments of the University of Cantabria approved the protocol (Permit Number: 2012/06) that follows the Spanish legislation (RD 1201/2005). All surgeries were performed under sodium pentobarbital anaesthesia, and all efforts were made to minimize suffering.

# Supplementary Figures and Tables

## Supplementary Figure 1


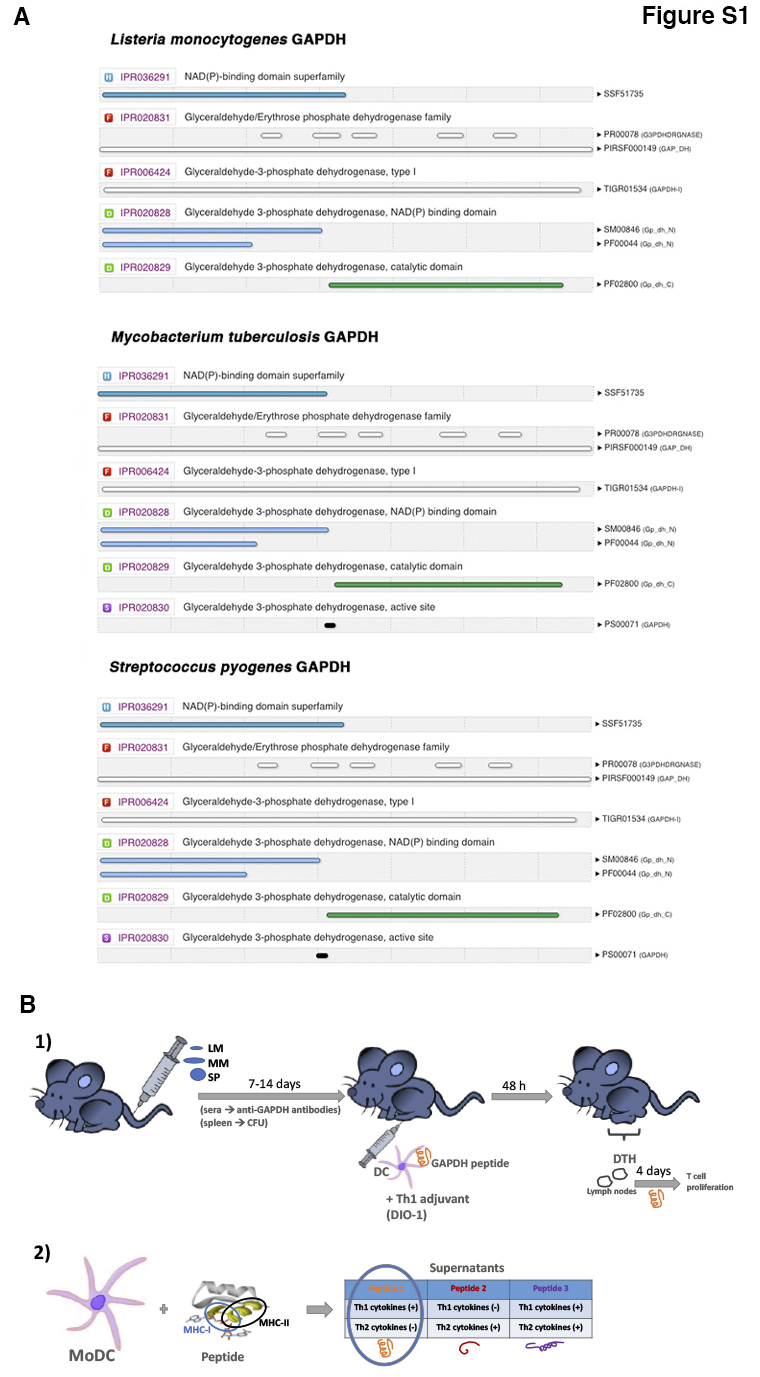


## Figure S1.- (*A*) InterPro analysis of GAPDH-LM, GAPDH-MTB and GAPDH-SP domains based on InterPro available at <https://www.ebi.ac.uk/interpro/protein/A0A121XBE7>, <https://www.ebi.ac.uk/interpro/protein/A0A045ITJ4> and <https://www.ebi.ac.uk/interpro/protein/P0C0G6>, that includes two entry types, family (red box) and domain (green box). The structural analysis of GAPDH-LM, GAPDH-MTB and GAPDH-SP proteins revealed the same two enzymatic domains for the three proteins: a NAD-binding domain at the N-terminal region defined by the Pfam entry PF00044 (light blue domains) and a C-terminal region involved in glycolysis that corresponds to the Pfam entry PF02800 (green domains). These proteins were positive for the same signatures and profiles of InterPro. (*B*) Model of approaches used in this study to select epitopes for multivalent vaccines. First approach *in vivo*, 1) mice are infected *i.v* (tail vain) with each of the bacterial isolates, LM, MM or SP. 7- or 14-days later, mice are bled to measure anti-GAPDH antibodies levels in sera and cytokines. A set of mice are sacrificed to check for CFU in spleens and another set are left alive and inoculated in the right hind footpads with dendritic cells loaded with the different GAPDH peptides in the presence of a Th1 adjuvant as DIO-1. 48 hours later, the swelling in the hint footpads is examined with a caliper to select the immunogenic GAPDH peptides as the strongest DTH reactions. Specific immune responses against GAPDH peptides were verified in removed popliteal lymph nodes that after cell homogenization and culture for 4 days in the presence or absence of the GAPDH peptide, confirmed those peptides inducing the strongest cellular immune responses as the best candidates for effective vaccines. In the second approach *in vitro* 2) monocyte derived dendritic cells (MoDC) from patients infected either with LM, MM or SP were loaded with GAPDH peptides of each bacteria, good MHC-I and MHC-II binders after the bioinformatic analysis. Examination of Th1 and Th2 cytokines in MoDC supernatants allows selection of those GAPDH peptides that induces mainly Th1 cytokines as the best candidates for effective vaccines.

## Supplemental Table S1. *In vivo* infection of mice selected clinical isolates from patients.

^a^Clinical isolates in the year 2016 from patients older than 50 years of age and infected with the following strains of *L. monocytogenes* (HUMV-LM01-HUMV-07, 7 isolates), different mycobacteria (M*. tuberculosis*, HUMV-MTB01 and *M. marinum*, HUMV-MM01 or HUMV-MM02) or different streptococci clinical isolates (*S. pneumoniae*, HUMV-SP01-HUMV-SP03) and selected from the 2014-2018 study. Non-pathogenic LM-∆LLO, *M. smegmatis* and *S. pneumoniae* 49619-19F strains were also included in the assay as non-virulent controls. ^b^Female C57BL/6 mice (n = 5) were *i.v.* inoculated with 5 x 10^3^ CFU bacteria from clinical isolates detailed in *a*. 14 days later, mice were bled, sacrificed and spleens collected. ^c^Sera from mice as in *b* were examined for anti-GAPDH-L1 antibodies by a peptide ELISA. Results are presented as the mean ± SD of OD units in triplicate experiments (*P* < 0.05). ^d^Spleens from mice as in *b* were homogenized and plated in blood agar plates. CFU were counted and results expressed as CFU/mL (*P* ≤ 0.5).

**
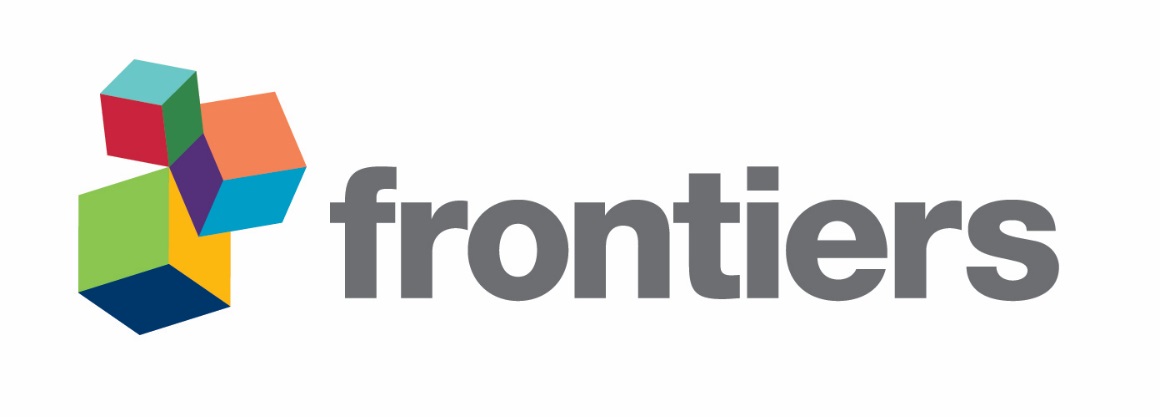
**
